# Structural Analyses of a Constitutively Active Mutant of Exchange Protein Directly Activated by cAMP

**DOI:** 10.1371/journal.pone.0049932

**Published:** 2012-11-26

**Authors:** Mark A. White, Sheng Li, Tamara Tsalkova, Fang C. Mei, Tong Liu, Virgil L. Woods, Xiaodong Cheng

**Affiliations:** 1 Sealy Center for Structural Biology and Molecular Biophysics, The University of Texas Medical Branch, Galveston, Texas, United States of America; 2 Department of Biochemistry and Molecular Biology, The University of Texas Medical Branch, Galveston, Texas, United States of America; 3 Department of Medicine and Biomedical Sciences Graduate program, University of California San Diego, La Jolla, California United States of America; 4 Department of Pharmacology and Toxicology, The University of Texas Medical Branch, Galveston, Texas, United States of America; Universite de Sherbrooke, Canada

## Abstract

Exchange proteins directly activated by cAMP (EPACs) are important allosteric regulators of cAMP-mediated signal transduction pathways. To understand the molecular mechanism of EPAC activation, we have combined site-directed mutagenesis, X-ray crystallography, and peptide amide hydrogen/deuterium exchange mass spectrometry (DXMS) to probe the structural and conformational dynamics of EPAC2-F435G, a constitutively active EPAC2 mutant. Our study demonstrates that conformational dynamics plays a critical role in cAMP-induced EPAC activation. A glycine mutation at 435 position shifts the equilibrium of conformational dynamics towards the extended active conformation.

## Introduction

**Figure 1:**
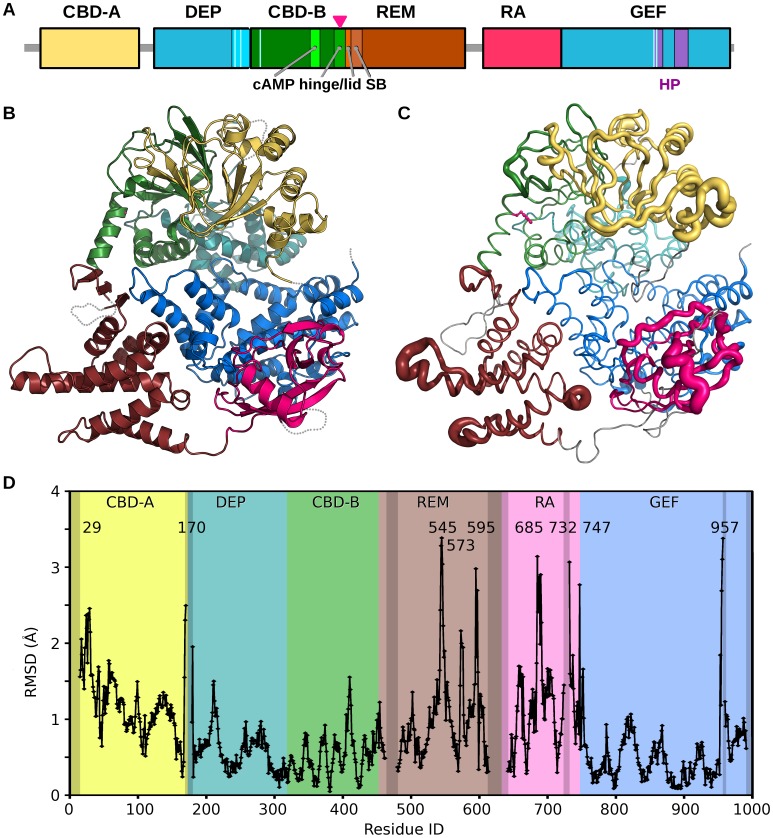
EPAC2-F435G Structure. (A) Schematic of the EPAC2 primary structure (yellow: CNBD-A; cyan: DEP; green: CNBD-B; brown: REM; red: RA; blue: GEF; red lines: ionic latch (IL); purple: receptor-binding Helical Hairpin (HP)). The magenta arrow indicates the point of mutation. (B) Crystal structure of EPAC2-F435G colored by domain as above with missing loops indicated by dotted gray lines. The site of mutation, F435G, is shown as a magenta ball. (C) Change in average Cα positions from apo-WT EPAC2 to apo-EPAC2-F435G shown as an RMSD worm. The diameter of the tube is proportional to the Cα-RMSD values. A narrow tube indicates a region with high structural similarity, while bulky tubes are regions which have moved more than the average. Missing or disordered regions are shown in gray, and the F435 side-chain in magenta ball-n-stick representation. (D) Change in Cα RMSD values from apo-EPAC2-F435G to apo-WT EPAC2 as a function residue number. The same domain color scheme is used for all Figures unless indicated otherwise.

Exchange proteins directly activated by cAMP (EPACs) are an important family of signaling molecules serving as the intracellular sensors for the prototypic second messenger [1], [2]. The two mammalian EPAC isoforms, EPAC1 and EPAC2, share extensive sequence and structural homology, which includes a conserved C-terminal catalytic core that consists of a RAS exchange (REM) domain, a RAS association (RA) domain, and a CDC25-homology guanine nucleotide exchange factor (GEF) domain. While both the N-terminal regulatory region of EPAC1 and EPAC2 contain a Dishevelled-Egl-Pleckstrin (DEP) domain and a cAMP binding domain (CBD), EPAC2 has an additional CBD in front of the DEP domain (Figure 1A). The physiological function of this extra CBD is not clear as it is not essential for the *in vitro* activity of EPAC2 [3]. The EPAC proteins exert their functions by acting as molecular switches in response to changes in cellular environments. When the intracellular concentration of cAMP rises, it binds to the cAMP binding domain (CBD) of EPAC and induces conformational changes, in the hinge and switchboard (SB), that lead to activation of EPAC by exposing the C-terminal catalytic core, which interacts with and activates down-stream effectors, Rap1 or Rap2 [1], [2] partially through residues in the Helical Hairpin (HP) [4].

Structure determinations of the full-length apo-EPAC2 and the ternary complex of an EPAC2 deletion construct in-complex with a cAMP analog and Rap1 have provided snapshots of the inactive and active conformations of EPAC2, respectively [5], [6]. While these three-dimensional structures have provided invaluable blueprints for unraveling the intricacies of the cAMP-induced activation process, our understanding of the molecular mechanism of EPAC activation is far from being complete as a crystal structure typically represents just one of the many possible low energy conformers in solution. Indeed, extensive molecular biophysics studies have confirmed that EPAC proteins exist, in solution, as a dynamic ensemble of multiple conformations [4], [7]–[14]. Therefore, insights into the conformational dynamics of EPAC are also essential. We have previously shown that mutations at position F435 can moderate the activity of EPAC2. A phenylalanine to glycine substitution at this position leads to a constitutively active EPAC2-F435G capable of activating down-stream effector Rap1 in the absence of cAMP with 60% of the WT EPAC activity under saturating level of cAMP [9]. In this study, we applied structural and molecular biophysical approaches to analyze the structure and dynamics of EPAC2-F435G, a constitutively active EPAC2 mutant with altered conformational dynamics.

## Materials and Methods

### Protein expression and purification

Recombinant full length wild-type EPAC2 and mutant EPAC2-F435G proteins were constructed, expressed and purified as previously described [9]. All proteins were at least 95% pure, as judged by SDS-polyacrylamide gel electrophoresis.

### Structure determination

Crystals of wild-type and mutant apo-EPAC2-F435G proteins were grown at 20°C in either sitting or hanging drops using reservoir solution containing 100 mM Bis-Tris Propane pH 7.5, 200 mM NaCl, 1.3 M (NH_4_)_2_SO_4_ and 6% glycerol. Crystals were soaked up to 30% glycerol in well solution for data collection. X-ray diffraction data, to 2.6 Å, were collected at SSRL synchrotron beamline 7.2 (Table 1). The EPAC2-F435G structure, which was in the same spacegroup and had similar cell dimensions to the published WT structure, was solved using rigid body refinement of the rebuilt 2BYV PDB entry [5] described below. Using CNS [15], [16] and PMB [17]–[19] each domain was placed as a rigid body, and then extensively rebuilt, including the flexible loops using composite omit maps. The stereochemical restraint optimization in PMB suggested a tight bond weight RMSD of 0.005 Å, which was used in refinement. This low X-ray weight along with the three sigma I/s(I) resolution of 3.0 Å, suggested that low-resolution refinement strategies should be applied, while retaining all the data to 2.6 Å. Therefore, in addition to the bond RMSD target of 0.005 Å, and the use of variable sigma-B restraints [20], weak residue-specific phi-psi restraints [21] were also included using PMB. These restraints are implemented in PMB using the CDIH, soft-well, dihedral angle restraints of CNS where the target angles are based on the residue type and location in the Molprobity Ramachandran regions [22]. Recalculation of the target region, at the beginning of refinement, and the use of only very weak energy terms permits residues to move readily between regions as indicated by the X-ray terms. The structure was manually rebuilt using COOT [23], and validated using Molprobity. The final model was compared to the extensively rebuilt and re-refined apo- and holo-EPAC2 structures, based on 2BYV and 3CF6, using PYMOL. The EPAC2-F435G mutant structure has been deposited with the PDB, pdb_id 4F7Z.

**Table 1:** Crystallographic Data Collection and Refinement.

(A) Data Collection	apo-Epac2 F435G	(B) Refinement	apo-Epac2 F435G
Beamline	SSRL BL 7-1	PDB-id	4F7Z
		Resolution (Å)	2.6
Space group	P 2_1_2_1_2_1_	Reflections	37,428
Cell Constants (Å)		Working Set Reflections	35,539
*a*	69.7	Test Set Reflections	1,889
*b*	95.3		
*c*	181.8	Rfactor (%)	27.2
		Rfree (%)	21.9
Resolution range	30 – 2.6		
(high resolution bin)	(2.66 – 2.60)	Number of atoms:	
		Protein atoms	7,506
Reflections (All)	147,255	Solvent molecules	81
Reflections (Unique)	37,453 (2,488)		
		Average B-factors (Å^2^)	
Multiplicity	3.9 (3.7)	Protein	78
Completeness (%)	98.5 (100)	Solvent	71
I/sigma (I)	34 (1.9)	Bond RMS (Å)	0.005
Rmerge (%)	4.7 (59)		
		Ramachandran Plot	
		Most Favored (%)	96.4
		Allowed Regions (%)	3.6
		Restricted (%)	0

### Models for MR and analysis

To provide models for the apo- and holo- conformations which were refined using identical procedures to the EPAC2-F435G structure the published crystal structures were rebuilt and refined. The incomplete models for the apo (2BYV) and holo- (3CF6) conformations of EPAC2 were extended to include missing residues using Swissmodel [24], [25]. These models were then refined, against the published Fobs, using PMB/CNS, and rebuilt manually, in COOT. Several cycles of refinement and rebuilding were able to extend some regions which were previously unmodeled and also produce reasonable models of several disordered loops. The complete 2BYV model was used for the initial rigid-body molecular replacement structure solution of the F435G mutant. Both models were used for comparison with the F435G structure.

### Deuterium Exchange Mass Spectrometry

Before carrying out hydrogen/deuterium exchange experiments, the optimal quench conditions that generate the best enzymatic cleaved peptide coverage maps of EPAC2 wt and EPAC2-F435G, were obtained as previously described [10], [26]. Functionally deuterated protein samples were prepared at 0°C by mixing 2.5 µl of stock solution of EPAC2 wt (3.5 mg/ml) or EPAC2-F435G (3.7 mg/ml) with 7.5 µl of deuterated buffer (8.3 mM Tris pD_read_ 7.2, 150 mM NaCl, 1 mM DTT and 1 mM EDTA in D_2_O) and incubating for 10, 100, 1000, 10000, 100000, and 1000000 sec. At the indicated time, the exchange reaction was quenched by addition of 15 µl of ice-cold optimal quench solution (0.8% formic acid, 16.6% glycerol and 1.6 M GuHCl), then the quenched samples were frozen on dry ice and stored at −80°C. Non-deuterated control samples were prepared in H_2_O buffer (8.3 mM Tris pH 7.2, 150 mM NaCl, 1 mM DTT and 1 mM EDTA in H_2_O) and equilibrium-deuterated samples (incubated in D_2_O buffer containing 0.5% formic acid for 1 day at 25°C) were also prepared. The samples were then thawed at 5°C and immediately passed over tandem protease columns (porcine pepsin, 16-µl bed volume, followed by *Aspergillus saitoi* fungal protease type XIII, 16 µl bed volume) with 0.05% trifluoroacetic acid (TFA) in water at a flow rate of 20 µl/min for 6 min. The proteolytic fragments were collected contemporaneously on a C18 Guard column (Michrom, Magic C18AQ, 0.2×2) and desalted for 1 min at 60 µl/min, then separated on a Michrom Reverse-phase C18 Column (Magic C18AQ, 3 µm, 200A, 0.2×50) with a linear gradient of acetonitrile from 6.4% to 38.4% over 30 min. The elutant was directed to a Finnigan LCQ Classic mass spectrometer with electrospray ionization voltage set at 4.5 kV, capillary temperature at 200°C, and data acquisition in either MS1 profile mode or data-dependent MS/MS mode. The SEQUEST software (Thermo Finnigan) was used for peptide identification and specialized software, DXMS Explorer (Sierra Analytics Inc.) was used to determine deuteration level of peptides in functionally deuterated samples as previously described [27], [28].

## Results

### X-ray crystal structure of apo-EPAC2-F435G

The crystal structure of apo-EPAC2-F435G, a constitutively active EPAC2 mutant, possesses similar cell dimensions to the published apo-EPAC2 structure, 2BYV [5]. This structure is known to correspond to the compact, inactive, form of EPAC2. The structure of EPAC2-F435G shares the tertiary and secondary structure of apo-EPAC2 (Figure 1B). The individual domains show little change from the WT structure. The domain-by-domain Cα-RMSDs between the two structures approach a resolution limited low value of 0.3 Å, while the overall Cα-RMSD reaches up to 1.2 Å. This difference is not entirely due to the flexibility of the 435-helix “hinge” between the N- and C-terminal regions, which rotates less than 6 degrees. Separately, the N- and C-terminal regions both show a similar Cα-RMSD of 0.8 Å. Gross structural changes due to the F435G mutation are observed for several loops, followed by moderate shifts in the CBD-A, REM and RA domains (Figure 1C). The Cα-RMSD (or difference distance plot) plot shows major peaks (>2 Å) at residues 29, 170, 545, 573, 595, 685, 732, 747, and 957 (Figure 1D). CBD-A has a high overall Cα-RMSD (1.2 Å) while there are only very minor structural changes in the DEP, CBD-B and switchboard regions, including the hinge region, and mutation site. This suggests a flexible linkage between the CBD-A and DEP domain. The REM domain displays shifts in the three helices from residue 545 to 613. The majority of the differences occur at the helix ends and the loops. The RA domain shows a large Cα-RMSD deviation (1.2 Å), with peaks at 685, 732 and 747. With the exception of a small region around residue 957, the CDC25 domain displays minimal structural differences (Figure 1C). Minor structural changes in the N-terminus include small motions in the DEP and CNBD-B domains, mostly in loop regions (Figure 1D).

**Figure 2:**
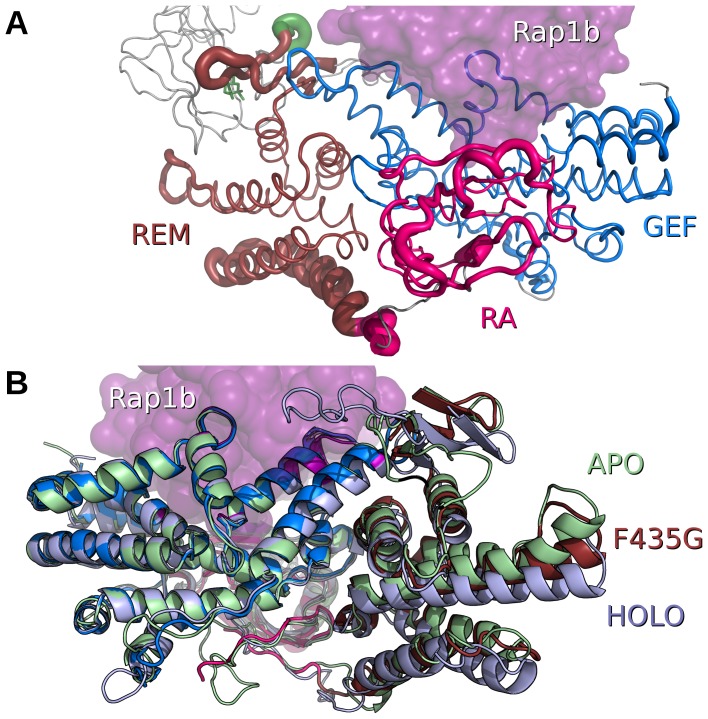
Superposition of the C-terminal catalytic region of apo-WT EPAC2, apo-EPAC2-F435G and holo-WT EPAC2. (A) RMSD worm drawing of apo-EPAC2-F435G/holo-WT EPAC2 colored by domain as in Figure 1B. The Rap1b molecule, bound to the holo-EPAC2 structure, is shown as a transparent, magenta-colored, solvent-accessible surface. The cAMP is shown as a green stick. (B) Crystal structure of apo-EPAC2-F435G, colored by domain, superpositioned on the apo-WT EPAC2 model, light-green and holo-WT EPAC2 model, light-blue. Alignment is based on the GEF domain, in blue. The Rap1b binding site is highlighted in purple. View is rotated 180° from that in A.

Since EPAC2-F435G is partially active in solution without ligand, we also compared its structure with the active holo-EPAC2 structure, 3CF6 (5). The N-terminal region of the holo-EPAC2 deletion structure includes only one domain, CBD-B, and it undergoes major structural changes at its C-terminal “activation switch” in response to cAMP binding in the holo-structure. So we focused our comparison on the C-terminal catalytic region. The overall Cα-RMSD value between the C-terminal domains of the EPAC2-F435G and holo-EPAC2 structures is low at 0.9 Å, slightly lower than between the C-terminal domains of the apo-WT and holo-EPAC2 structures (1.1 Å). There are exceptions, and these occur in the RA and REM domains where an increase in disordering of the turn between REM helices 578–594 and 599–613 is observed between holo-EPAC and apo-EPAC2-F435G structure (Figure 2A). The start of helix 545–571 in the REM domain displays a bending motion in the apo-WT structure. The F435G mutant is intermediate between the apo-WT and holo structures, but more similar to the holo-conformation. The motion at the tips of the helices corresponds to an RMSD of 3.4 Å to apo and 1.9 Å to holo, for a total shift of 3.6 Å (Figure 2B).

### Changes in structural dynamics as measured by B-factors

**Figure 3:**
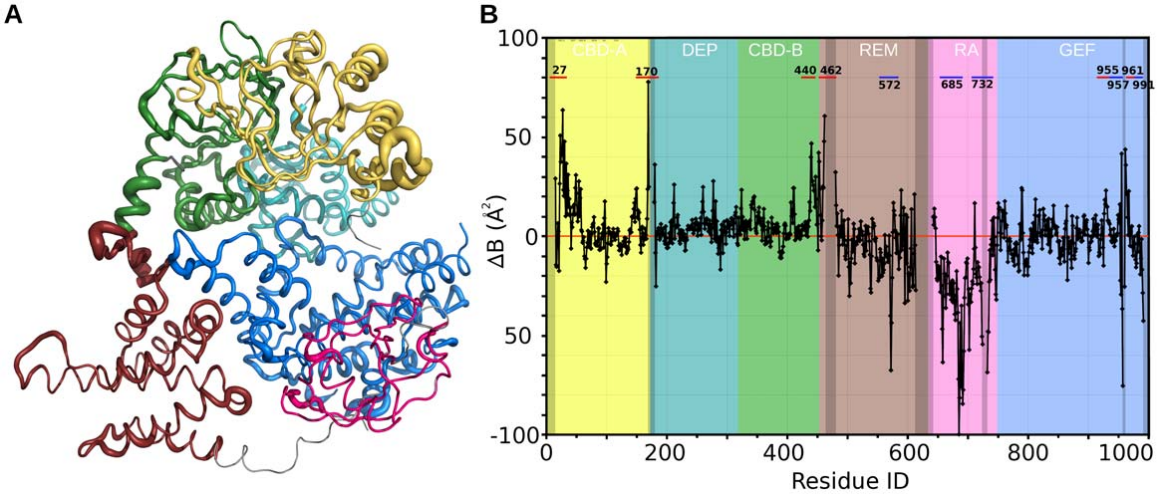
Relative changes in B-factors from apo-EPAC2 to EPAC2-F435G. (A) Relative changes in B-factors are shown as a ΔB-factor worm. The diameter of the tube is proportional to the difference in B-factors in each structure relative to the average. Coloring is the same as in Figure 1C. (B) Plot of ΔB-factor (offset by average B). Background is colored by domain as in A. Missing regions are grayed-out. The residue numbers of the largest difference peaks are labeled.

In addition to the aforementioned structural differences, the effect of the Phe to Gly mutation at residue 435 is most strongly presented in the change in the dynamics of the system, correspond to large changes in the B-factors between the two structures (Figure 3A). Overall, the N-terminal regulatory region of the F435G mutant is more dynamic, particularly at the N-terminus (residues 23–30), the CBD-A/DEP linker (residue 169), and the hinge/switchboard region (residues 439–462). Most interestingly, while the hinge, a turn C-terminal to the mutation, and part of the switchboard region showed minimal structural changes (Figure 1C) they displayed the greatest increases in B-factors (Figure 3A). On the other hand, the C-terminal catalytic region has greater stability in the REM (residue 572), the RA and the CDC25 (residue 957) domains. While the EPAC2-F435G REM and RA domains appear to be relatively more stable as compared to those of the WT EPAC2, they were already highly dynamic, and are still the most dynamic domains with the highest average B-factors (Figure 3B) and significant internal motions (Figure 1C).

### DXMS analyses of wild-type EPAC2 and EPAC2-F435G mutant protein

We applied DXMS technique to further examine and compare the conformational dynamics of wild-type and mutant EPAC2 proteins in solution. Tandem protease digestion of the mutant F435G protein provided a similar but distinct digestion pattern as compared to that of the wild-type (WT) protein (Figure S1). Overall, a total of 332 or 386 peptide fragments were observed for the WT EPAC2 or EPAC2-F435G mutant, respectively. Among these peptide fragments, 276 of them are common between the WT EPAC2 and EPAC2-F435G mutant. These 276 peptide fragments span a majority of the EPAC sequence. In fact, for areas with multiple peptide fragments observed in both the WT and mutant, only one region (422–446), as highlighted by a box in the Figure S1, does not contain any commonly matched fragments between the WT EPAC2 and EPAC2-F435G mutant. This observation is consistent with the fact that this region immediately encompasses the site of the F435G mutation.

**Figure 4:**
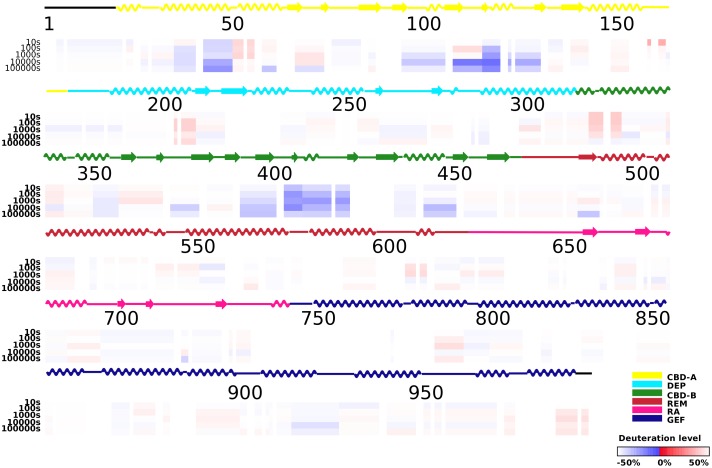
Changes in hydrogen/deuterium exchange rates between the apo-EPAC2-F435G and apo-WT EPAC2. (A) Differences in deuteration levels between the apo-EPAC2-F435G and **apo-WT EPAC2** at various time points (from top to bottom: 10, 30, 100, 1,000, 10,000, and 100,000 seconds) are shown in color-coded bars ranging from blue (−50%) to red (50%), as indicated in the bottom-right corner of the figure. The site of mutation is indicated by a magenta arrow. (B) Changes in the percent of deuterium incorporation by the F435G mutation for common individual peptides, between the apo-EPAC2-F435G and WT after 1000 s incubation in D_2_O buffer, are shown as bars spanning the indicated sequence along the x-axis. Top: domain color scheme of EPAC2 structure.

**Figure 5:**
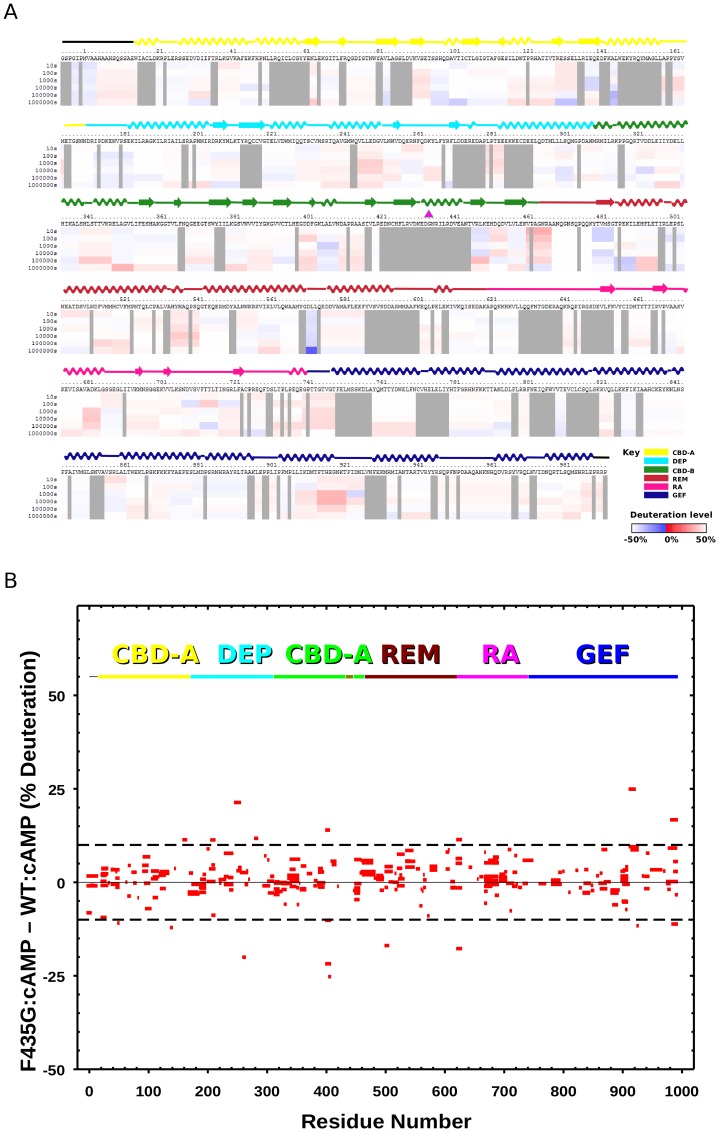
Changes in hydrogen/deuterium exchange rates between cAMP-bound EPAC2-F435G and cAMP-bound WT EPAC2. (A) Differences in deuteration levels between the cAMP-bound EPAC2-F435G and **cAMP-bound WT EPAC2** at various time points (from top to bottom: 10, 30, 100, 1,000, 10,000, and 100,000 seconds) are shown in color-coded bars ranging from blue (−50%) to red (50%), as indicated at the bottom-right corner of the figure. The site of mutation is indicated by a magenta arrow. (B) Changes in the percent of deuterium incorporation by the F435G mutation for common individual peptides, between the cAMP-bound EPAC2-F435G and **cAMP-bound WT EPAC2** after 1000 s incubation in D_2_O buffer, are shown as bars spanning over the indicated sequence along the x-axis. Top: domain color scheme of EPAC2 structure.

When the H/D exchange rates of the 276 commonly matched peptide fragments of the WT EPAC2 and EPAC2-F435G mutant proteins in the absence of cAMP were compared (Figure S2), an overall increase in the level of deuteration was observed for EPAC2-F435G mutant spanning the entire sequence of EPAC2 (Figure 4A & B). This enhanced rate of H/D exchange is particularly apparent for areas adjacent to either side of the hinge. A similar increase in the levels of deuteration for peptides within the hinge, at the gap (422–446) where there are no matched peptide fragments, was also observed (Figure S3). On the other hand, the difference in H/D exchange rates of the 276 commonly matched peptides was much smaller in the presence of cAMP, with an even distribution of both increases and decreases in exchange rates (Figure 5A & B).

**Figure 6:**
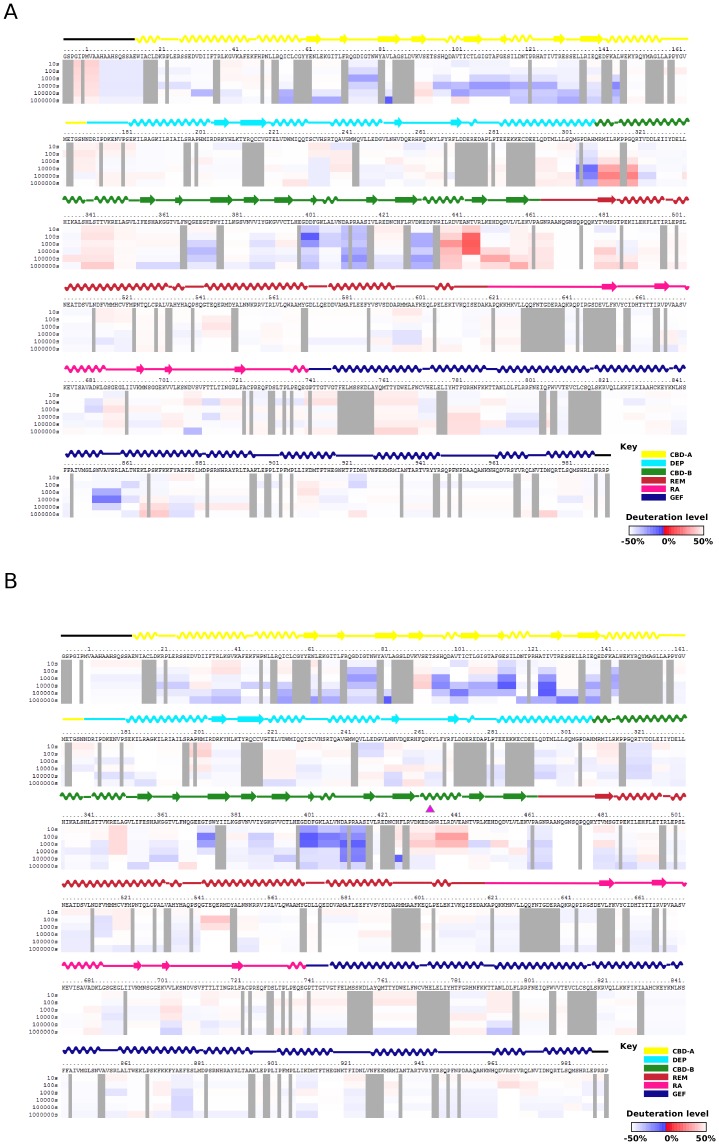
Changes in hydrogen/deuterium exchange rates of EPAC2 induced by binding of cAMP. Differences in deuteration levels in the free and cAMP-bound WT EPAC2 (A) or in the free and cAMP-bound EPAC2-F435G (B) at various time points (from top to bottom: 10, 30, 100, 1,000, 10,000, and 100,000 seconds) are shown as color-coded bars range from blue (−50%) to red (50%), as indicated at the bottom-right corner of the figure. The magenta arrow indicates the point of mutation.

As revealed by our earlier DXMS study [10], the most noticeable conformational change induced by cAMP in the wild-type EPAC2 protein is at the hinge region as demonstrated by a large increase in the rate of H/D exchange (Figure 6A, Figure S4A). While an enhanced rate of H/D exchange was also observed for the EPAC2-F435G mutant protein in response to cAMP, the magnitude of the changes is noticeably reduced in the mutant protein (Figure 6B, Figure S4B). This difference is due to the fact that the hinge region in apo-EPAC2-F435G mutant is significantly more dynamic and/or solvent accessible than that of the apo-EPAC2 as discussed above.

## Discussion

A Phe to Gly mutation at residue 435 within the critical hinge region of EPAC leads to a constitutively active protein without perturbing its cAMP binding function, suggesting that even in the absence of cAMP a significant fraction of the EPAC2-F435G protein exist in the active conformation [9]. In contrast, WT EPAC2 activation requires the binding of cAMP. While the crystal structure of apo-WT EPAC2 captures a compact (closed), auto-inhibited conformation [5], the cAMP-bound structure of a truncated EPAC2 shows an extended (open) form in complex with Rap1b [7]. However, this deletion EPAC2-Rap1b crystal structure is not necessarily the same as the un-complexed solution structure of the full-length EPAC2 active form, which has been refractory to crystallization. X-ray crystallographic analysis of the apo-EPAC2-F435G mutant reveals several prominent features when compared to the previous WT Epac2 crystal structures. First of all, even though EPAC2-F435G is active in solution without cAMP, the apo-EPAC2-F435G crystal structure still represents the compact, inactive apo form of EPAC2, trapped by the crystal lattice, which is incompatible with the extended, active conformation. Second, while structural changes immediately adjacent to the site of mutation between WT and EPAC2-F435G are relatively small, major structural deviations occur at distal sites, particularly at the C-terminal catalytic lobe, suggesting global allosteric effects of the mutation (Figure 1). Third, part of the C-terminal catalytic region of EPAC2-F435G is more similar to the active holo-conformation than to the apo-EPAC2 (Figure 2B). Fourth, the EPAC2-F435G protein, especially the N-terminal regulatory lobe, is more dynamic overall than its WT counterpart in the crystal structure as indicated by an increase in average domain B-factors, with the exception of the RA domain. Last, while almost all the sites with major changes in B-factor show significant RMSD alterations from the previous crystal structures (Figures 1C & 3B), one region, the hinge/switchboard (residues 439–462) stands out: it showed the largest increases in B-factors but exhibited little structural perturbation. This apparent disparity between changes in structure and dynamics suggests that a significant number of constrains are placed on the hinge, like a loaded spring, when EPAC2-F435G proteins are held in the inactive apo conformation within the crystal lattice, a graphic indication of the destabilization of the hinge by the F435G mutation. Results obtained by DXMS also show that the F435G mutation causes the largest change in dynamics in this region when the protein is in solution (Figure 4). Taken together, our structural analysis reveals that the F435G mutation results in significant inter-domain allosteric flexibility and increases the conformational dynamics of the activation switch in the apo-conformation.

Consistent with X-ray crystallographic analyses, our DXMS studies further confirm that EPAC2-F435G is overall more dynamic in solution, particularly in the hinge/switchboard region. From a comparison of the apo- and holo-EPAC2 structures it is observed that during EPAC activation the C-terminal end of hinge helix (432–445) melts and that the REM β-sheet of the “switchboard” rotates to form one side of the cAMP binding pocket, the side blocked by the CBD-A binding pocket in the apo-WT EPAC2 structure [5], [6]. Thus, based on both our structural and hydrogen exchange studies, it appears that the immediate effect of the F435G mutation is on the EPAC2 activation switch. As a consequence, the increased flexibility around the hinge/switchboard lowers the activation barrier between the inactive intermediate and active conformations, shifting the conformational dynamics of apo-EPAC2-F435G toward the active states, resulting in a constitutively active mutant. It will be interesting to test if binding of recently discovered EPAC specific inhibitors [29]–[31] would block this shift in conformational dynamics.

## Supporting Information

Figure S1 **Digestion maps of EPAC2 and EPAC2-F435.** Peptide fragmentation pattern (indicated by the solid lines: Cyan, WT only; Red, EPAC2-F435G only; Blue, shared) of cAMP-free EPAC2. The secondary structures of EPAC2 are shown above the peptide fragments and are colored by domain: Yellow: CBD-A, Cyan: DEP, Green: CBD-B, Brown: REM, Red: RA, Blue: GEF. Box indicates region with no overlap peptides between EPAC2-F435G and WT-EPAC2. The site of the mutation is marked by a magenta arrow.
(TIF)

Figure S2 **Summary of hydrogen/deuterium exchange rates of apo-WT EPAC2 and apo-EPAC2-F435G.** Deuteration levels of representative peptide fragments of apo-WT EPAC2 (A) and apo-EPAC2-F435G (B) at various time points (from top to bottom: 10, 100, 1,000, 10,000, and 100,000 seconds) are shown as a pseudo color scale. The site of the F435G mutation is marked by a magenta arrow.
(TIF)

Figure S3 **Comparison of hydrogen/deuterium exchange rates between apo-EPAC2-F435 and apo-EPAC2.** Percent of deuterium incorporation for uniquely identified individual peptides of the apo-EPAC2-F435G (black) and apo-EPAC2 (red) between residues 420–450 after 1000 s incubation in D_2_O buffer are shown as bars spanning over the indicated sequence on the x-axis.
(TIF)

Figure S4 **Summary of hydrogen/deuterium exchange rates of EPAC2 in the absence and presence of ESI-07.** Deuteration levels of representative peptide fragments of EPAC2 alone (A) and EPAC2-ESI-07 complex (B) at various time points (from top to bottom: 10, 100, 1,000, 10,000, and 100,000 seconds) are shown as a pseudo color scale. The site of the F435G mutation is marked by a magenta arrow.
(TIF)
