# Supplementary figures and images for: Structural Analyses of a Constitutively Active Mutant of Exchange Protein Directly Activated by cAMP
Source: PLoS One. 2012 Nov 26;7(11):e49932. doi: 10.1371/journal.pone.0049932 (PMC3506601; doi:10.1371/journal.pone.0049932)

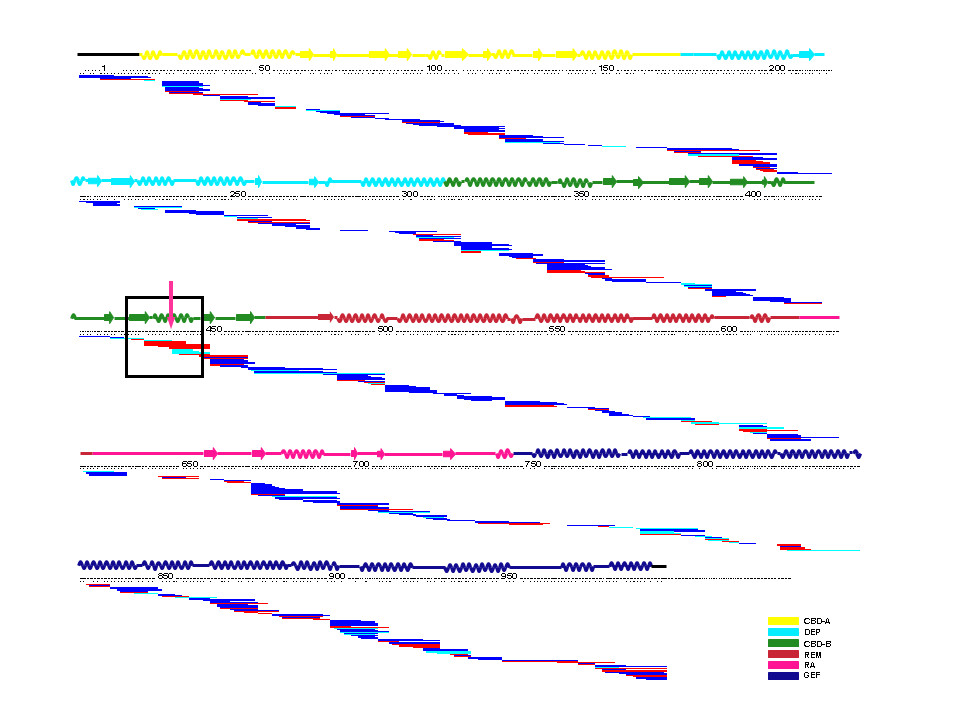

Supplement: Figure S1 — Digestion maps of EPAC2 and EPAC2-F435. Peptide fragmentation pattern (indicated by the solid lines: Cyan, WT only; Red, EPAC2-F435G only; Blue, shared) of cAMP-free EPAC2. The secondary structures of EPAC2 are shown above the peptide fragments and are colored by domain: Yellow: CBD-A, Cyan: DEP, Green: CBD-B, Brown: REM, Red: RA, Blue: GEF. Box indicates region with no overlap peptides between EPAC2-F435G and WT-EPAC2. The site of the mutation is marked by a magenta arrow. (TIF) [file pone.0049932.s001.tif]

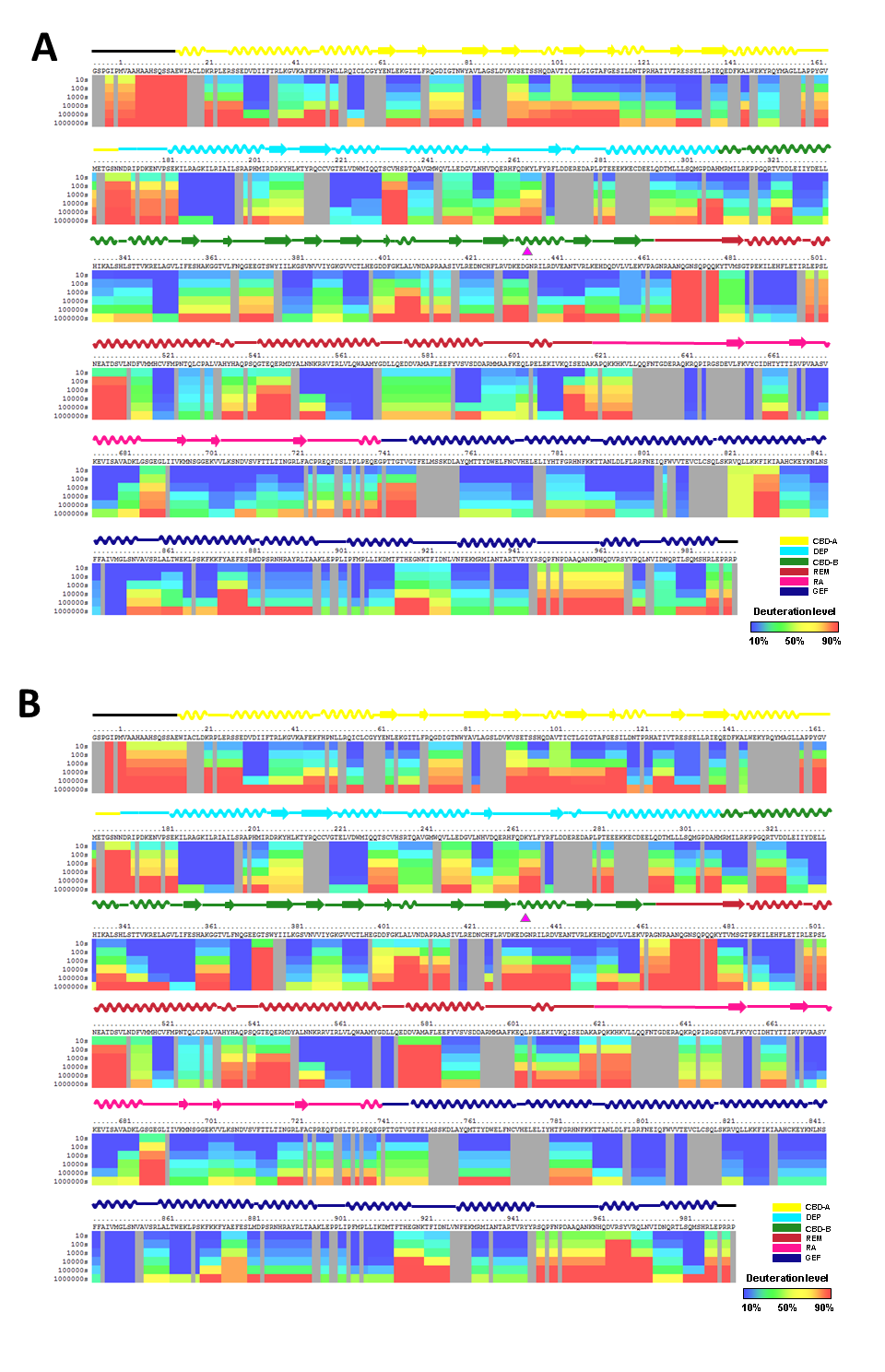

Supplement: Figure S2 — Summary of hydrogen/deuterium exchange rates of apo-WT EPAC2 and apo-EPAC2-F435G. Deuteration levels of representative peptide fragments of apo-WT EPAC2 (A) and apo-EPAC2-F435G (B) at various time points (from top to bottom: 10, 100, 1,000, 10,000, and 100,000 seconds) are shown as a pseudo color scale. The site of the F435G mutation is marked by a magenta arrow. (TIF) [file pone.0049932.s002.tif]

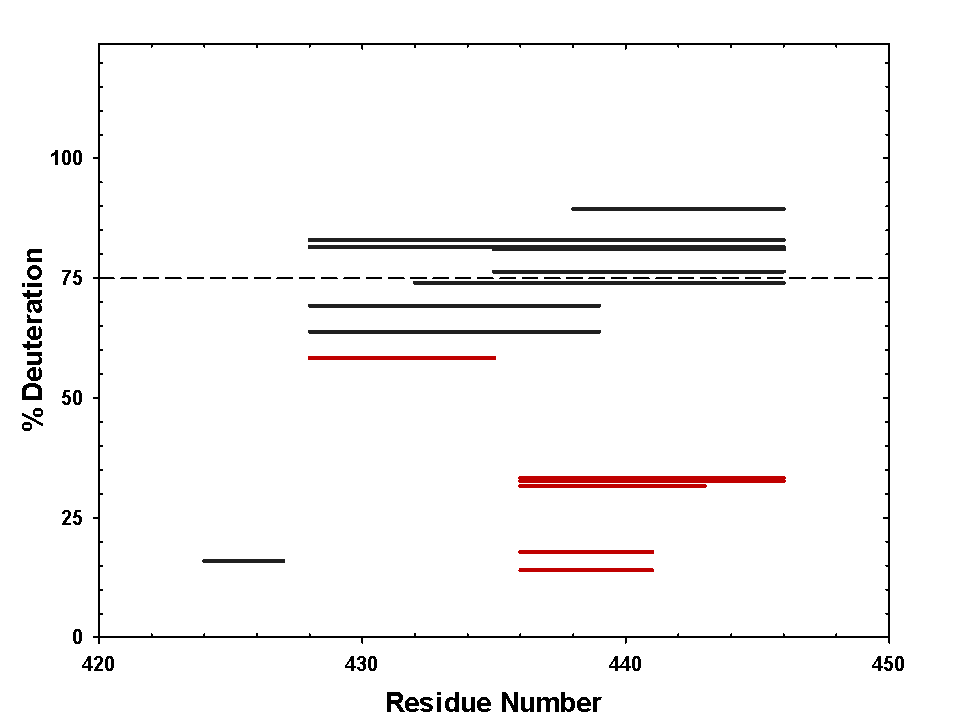

Supplement: Figure S3 — Comparison of hydrogen/deuterium exchange rates between apo-EPAC2-F435 and apo-EPAC2. Percent of deuterium incorporation for uniquely identified individual peptides of the apo-EPAC2-F435G (black) and apo-EPAC2 (red) between residues 420–450 after 1000 s incubation in D2O buffer are shown as bars spanning over the indicated sequence on the x-axis. (TIF) [file pone.0049932.s003.tif]

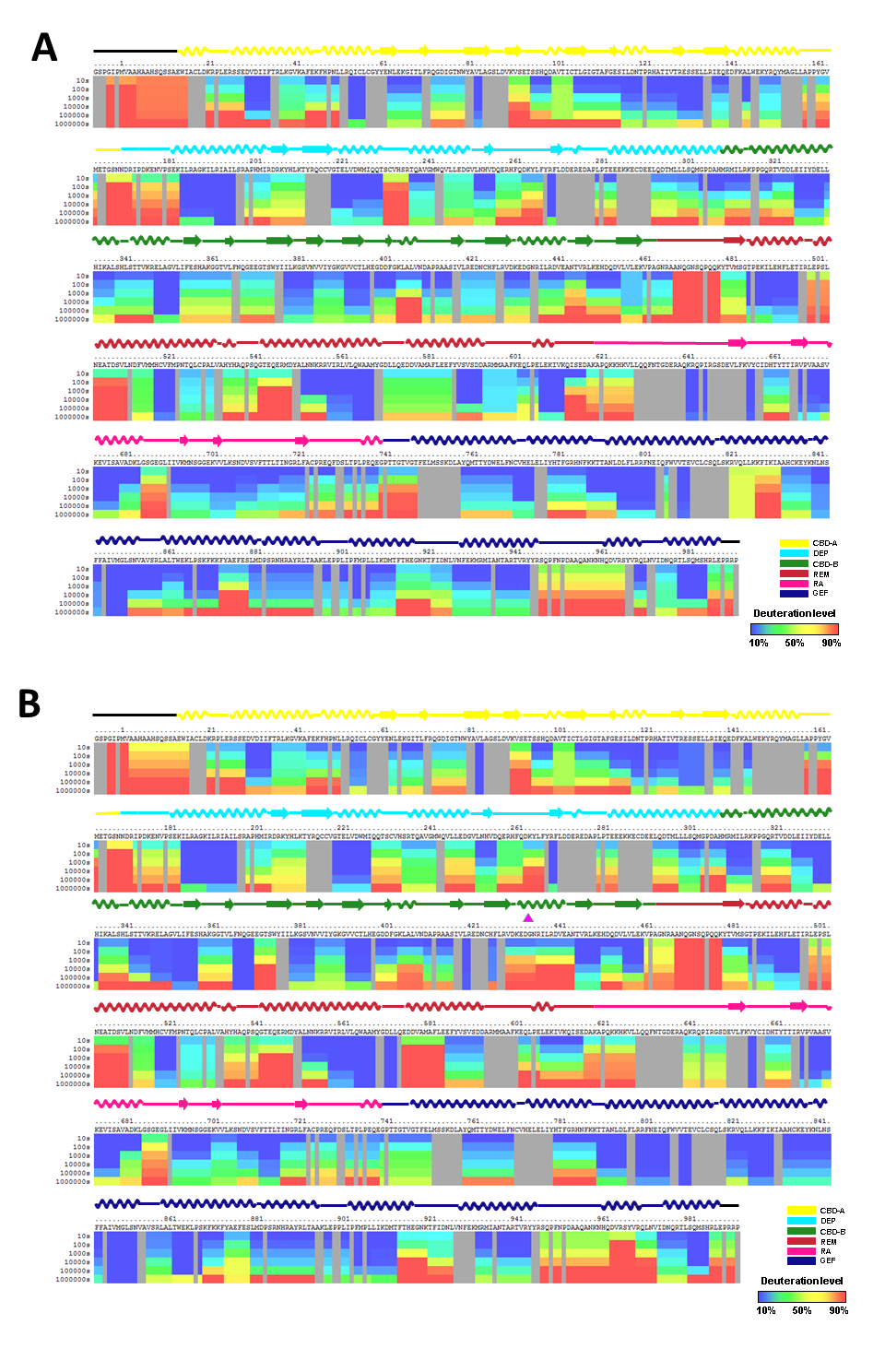

Supplement: Figure S4 — Summary of hydrogen/deuterium exchange rates of EPAC2 in the absence and presence of ESI-07. Deuteration levels of representative peptide fragments of EPAC2 alone (A) and EPAC2-ESI-07 complex (B) at various time points (from top to bottom: 10, 100, 1,000, 10,000, and 100,000 seconds) are shown as a pseudo color scale. The site of the F435G mutation is marked by a magenta arrow. (TIF) [file pone.0049932.s004.tif]
